# Assessment of causal associations among gut microbiota, metabolites, and celiac disease: a bidirectional Mendelian randomization study

**DOI:** 10.3389/fmicb.2023.1087622

**Published:** 2023-05-12

**Authors:** Ting Li, Yan Feng, Chun Wang, Tian Shi, Adilai Abudurexiti, Mengxia Zhang, Feng Gao

**Affiliations:** ^1^Department of Gastroenterology, People’s Hospital of Xinjiang Uygur Autonomous Region, Urumqi, Xinjiang, China; ^2^Department of Pathology, People’s Hospital of Xinjiang Uygur Autonomous Region, Urumqi, Xinjiang, China; ^3^Xinjiang Clinical Research Center for Digestive Diseases, Urumqi, Xinjiang, China

**Keywords:** celiac disease, gut microbiota, metabolites, Mendelian randomization analysis, causal effect

## Abstract

**Background:**

A growing number of studies have implicated that gut microbial abundance and metabolite concentration alterations are associated with celiac disease (CD). However, the causal relationship underlying these associations is unclear. Here, we used Mendelian randomization (MR) to reveal the causal effect of gut microbiota and metabolites on CD.

**Methods:**

Genome-wide association study (GWAS) summary-level data for gut microbiota, metabolites, and CD were extracted from published GWASs. Causal bacterial taxa and metabolites for CD were determined by two-sample MR analyses. The robustness of the results was assessed with sensitivity analyses. Finally, reverse causality was investigated with a reverse MR analysis.

**Results:**

Genetically, increased genus *Bifidobacterium* was potentially associated with higher CD risk (odds ratio [OR] = 1.447, 95% confidence interval [CI]: 1.054–1.988, *p* = 0.022) while phylum *Lentisphaerae* (OR = 0.798, 95% CI: 0.648–0.983, *p* = 0.034) and genus *Coprobacter* (OR = 0.683, 95% CI: 0.531–0.880, *p* = 0.003) were related to lower CD risk. Moreover, there were suggestive associations between CD and the following seven metabolites: 1-oleoylglycerophosphoethanolamine, 1-palmitoylglycerophosphoethanolamine, 1,6-anhydroglucose, phenylacetylglutamine, tryptophan betaine, 10-undecenoate, and tyrosine. Sensitivity analyses deemed the results reliable without pleiotropy.

**Conclusion:**

We investigated the causal relationships between gut microbiota, metabolites, and CD with two-sample MR. Our findings suggest several novel potential therapeutic targets for CD treatment. Further understanding of the underlying mechanism may provide insights into CD pathogenesis.

## 1. Introduction

Celiac disease (CD) is characterized by an immune-mediated enteropathy that affects the small intestine (Green and Cellier, 2007; Lebwohl et al., 2018). In predisposed individuals, gluten protein ingestion induces villous atrophy in the small bowel mucosa with lymphocyte infiltration. A systematic review noted that CD has a global prevalence of 1.4% and varies from 1.3 to 1.8% in different continents (Singh et al., 2018). Individuals carry the risk alleles [human leukocyte antigen (HLA)-DQ2, HLA-DQ8, and HLA-DQ7] and gluten ingestion triggering is necessary but insufficient for CD development (Fasano and Catassi, 2001). The gut microbiota and specific metabolites are considered cofactors in CD pathophysiology (Caminero et al., 2019). Therefore, investigating the interaction between host genetics and gut microbiota or metabolites is likely important in CD pathogenesis.

Numerous recent studies have indicated that intestinal flora changes are tightly correlated with autoimmune diseases (Chen et al., 2017; De Luca and Shoenfeld, 2019). As a key regulator of the gastrointestinal tract, gut bacteria influence the synthesis of many nutrients *via* insoluble fiber digestion, vitamin production, and bile acid metabolism (Olivares et al., 2011; Caminero et al., 2014; Valdes et al., 2018). The gut microbiota regulate gluten protein digestion, which can affect antigen development. Furthermore, human intestinal microbes produce a wide variety of metabolites, which act in the bloodstream and exert systemic effects on humans. Alterations in gut microbiota or metabolites can lead to biological changes in diseases, indicating that they might be causes and treatment targets of these diseases (Vacca et al., 2022; Zoghi et al., 2022).

Although links between gut microbiota and risk of CD development have been established based on observational studies, the causal nature of these links remains poorly understood. Some recent studies investigated the causal relationship between gut microbiota and autoimmune disease with a two-sample Mendelian randomization (MR) analysis (Xu et al., 2021; Zhang et al., 2021). MR analysis is an instrumental variable (IV) approach aimed at inferring a causal relationship between an exposure and an outcome from observational studies (Lee and Lim, 2019). Two-sample MR analysis is a major extension and improvement of the MR method. Genetic variants are associated with exposure as IVs to quantify causal relationships between the exposures and outcomes.

Here, we used a two-sample MR approach to verify the causal relationships among gut microbiota, metabolites, and CD. We identified several genetic variants linked to the bacterial composition that may drive CD pathogenesis. Our findings could form the basis for developing new research lines for diagnosing and treating CD.

## 2. Methods

### 2.1. Study design

**Figure 1:**
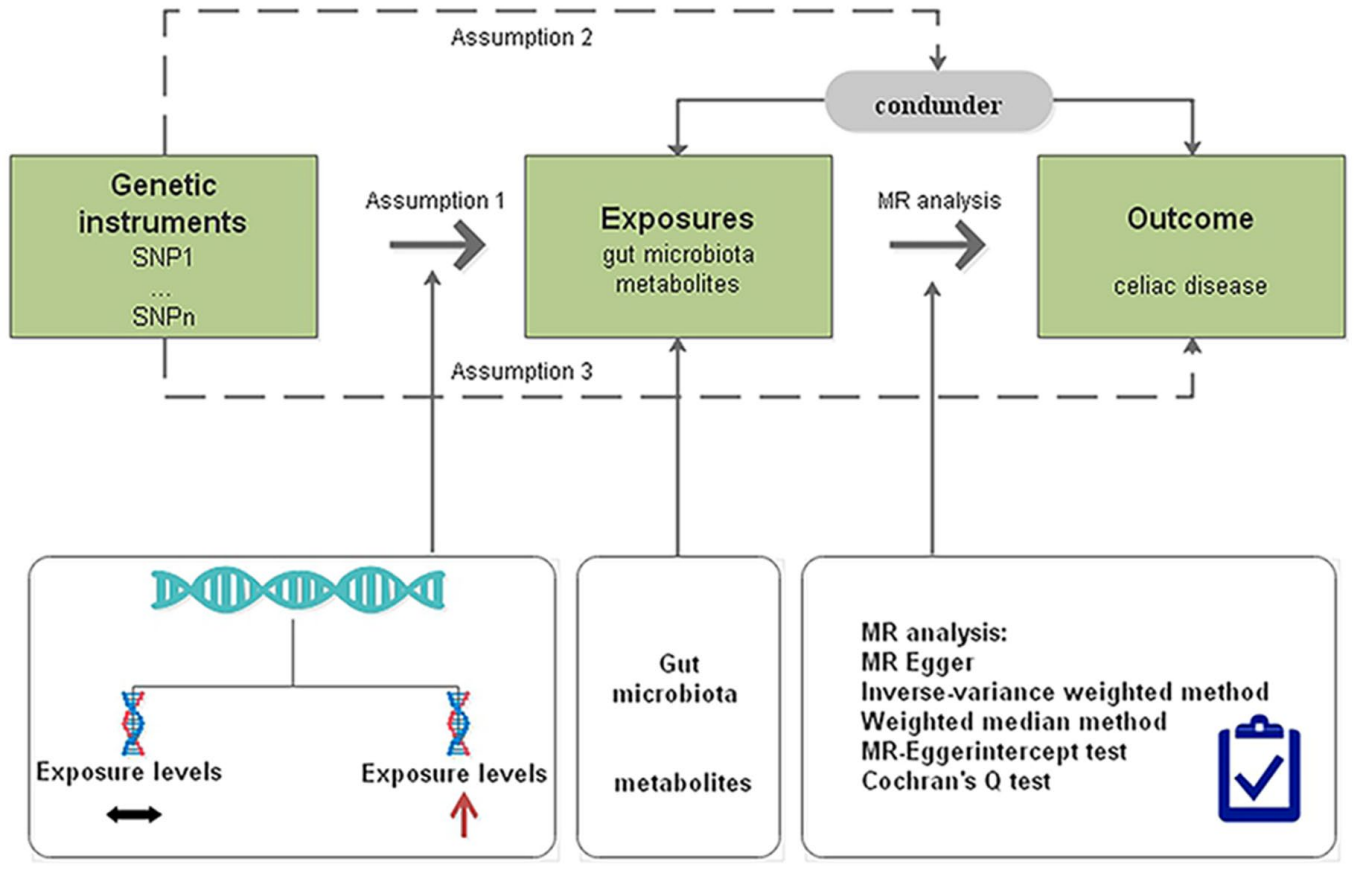
Diagrammatic description of MR analysis. SNP, single-nucleotide polymorphism; MR, Mendelian randomization.

We determined the causal effects of gut microbiota and metabolites on CD through MR analysis with genome-wide association study (GWAS) summary data. The MR design should meet three prerequisites (Figure 1): (i) the genetic variant selected as the IV is associated with the gut microbiota and metabolites; (ii) the genetic instruments are independent of potential confounders; and (iii) the genetic variant is associated with CD only through gut microbiota and metabolites but not other pathways (Boef et al., 2015).

### 2.2. Gut microbiota data sources

The MiBioGen consortium recruited 18,340 individuals with diverse ethnic backgrounds, including European, Latin/American Hispanic, and East Asian ethnicities (Kurilshikov et al., 2021). The summary statistics yielded the most comprehensive demonstration of genetic influences on human gut microbiota to date. The microbiome quantitative trait loci (mbQTL) mapping analysis included only the taxa that were present in at least 10% of the samples, which totaled 211 taxa and included nine phyla, 16 classes, 20 orders, 35 families, and 131 genera. The complete statistics of the association study are available at the www.mibiogen.org website. In the present study, we removed 15 bacterial traits without specific names, leaving 196 bacterial traits for further analysis (9 phyla, 16 classes, 20 orders, 32 families, and 119 genera).

### 2.3. Metabolite data sources

As metabolites are integral in gut microbiota–host crosstalk, we analyzed the GWAS summary data of the human metabolome involving 7,824 participants. A total of 486 metabolite concentrations were tested in the GWAS (Shin et al., 2014). The GWAS data used in this study are free to access at https://metabolomics.helmholtz-muenchen.de/gwas/index.php?task=download.

### 2.4. Instrumental variable selection

The IVs were screened with the criterion *p* < 1 × 10^−5^ to obtain a more comprehensive result. Next, all IVs underwent linkage disequilibrium (LD) clumping (*r*^2^ = 0.01; distance = 10,000 kb) to reduce the influence of correlations between single nucleotide polymorphisms (SNPs). SNPs with inconsistent alleles between the exposure and outcome samples and palindromic SNPs with intermediate allele frequencies were all removed. The strength of the selected SNPs was evaluated using the F-statistic.

### 2.5. Celiac disease data sources

In the present study, we used the largest GWAS on CD, which was published by Dubois and colleagues in 2010. GWAS summary data were extracted from the Integrative Epidemiology Unit (IEU) GWAS database. Dubois et al. genotyped a total of 500,000 SNPs from 4,533 CD patients and 10,750 controls (Dubois et al., 2010).

### 2.6. Mendelian randomization analysis

After the eligible IVs had been selected, MR analysis was conducted to determine the causal relationship between gut microbiota, metabolites and CD risk. The causal effect was examined with complementary approaches such as MR-Egger, inverse-variance weighted (IVW), simple and weighted modes, and weighted median. The results are mainly based on the IVW method, which was complemented by the other four approaches. Multiple hypothesis test correction was performed using the bonferroni correction. Bonferroni-corrected *p* < 0.05 indicated a significant association while *p* < 0.05 but Bonferroni-corrected *p* > 0.05 indicated a suggestive evidence of association. Power calculations were conducted based on the mRnd website^1^ (Brion et al., 2013). For significant estimates, we appraised horizontal pleiotropy based on the intercept term derived from MR-Egger regression. The pleiotropic biases were tested with MR-PRESSO and the pleiotropic effects were corrected by removing the outliers. We estimated the statistical heterogeneity of the IVW meta-analysis using Cochran’s Q statistics. All analyses were performed using the R package TwoSampleMR v0.5.6 (R version 4.1.2).

### 2.7. Reverse Mendelian randomization analysis

To investigate if CD had any causal effects on any gut microbiota abundance or metabolite concentrations, we performed a reverse MR analysis (CD as exposure and gut microbiota and metabolites as outcomes) using CD-associated SNPs as IVs. MR analysis was conducted as described earlier.

## 3. Results

### 3.1. Selection of instrumental variables

The F-statistics for the selected IVs were 11.08–95.33 and all reached the threshold of >10, indicating that the causal estimations did not have weak-instrument bias. The MR-PRESSO global test detected no evidence of pleiotropic effects (*p* > 0.05). The Supplementary Table S1 lists the association results between the bacterial traits and CD risk. 48 relevant SNPs associated with gut microbiota characteristics were selected as IVs with a series of quality-control steps. Specifically, 7 independent SNPs were associated with phylum *Lentisphaerae*, 8 independent SNPs were associated with order *Bifidobacteriales* for CD, 8 independent SNPs were associated with family *Bifidobacteriaceae* for CD, 9 independent SNPs were associated with genus *Bifidobacterium*, 8 independent SNPs were associated with genus *Coprobacter*, and 8 independent SNPs were associated with genus *Subdoligranulum*.

Regarding metabolites, 7 independent SNPs were associated with 1-oleoylglycerophosphoethanolamine for CD, 29 independent SNPs were associated with 1-palmitoylglycerophosphoethanolamine, 8 independent SNPs were associated with 1,6-anhydroglucose, 31 independent SNPs were associated with 10-undecenoate, 17 independent SNPs were associated with phenylacetylglutamine (PAGln), 11 independent SNPs were associated with tryptophan betaine, and 28 independent SNPs were associated with tyrosine. IVs used are listed in the Supplementary Table S4.

### 3.2. Causal effects of gut microbiota on celiac disease

**Figure 2:**
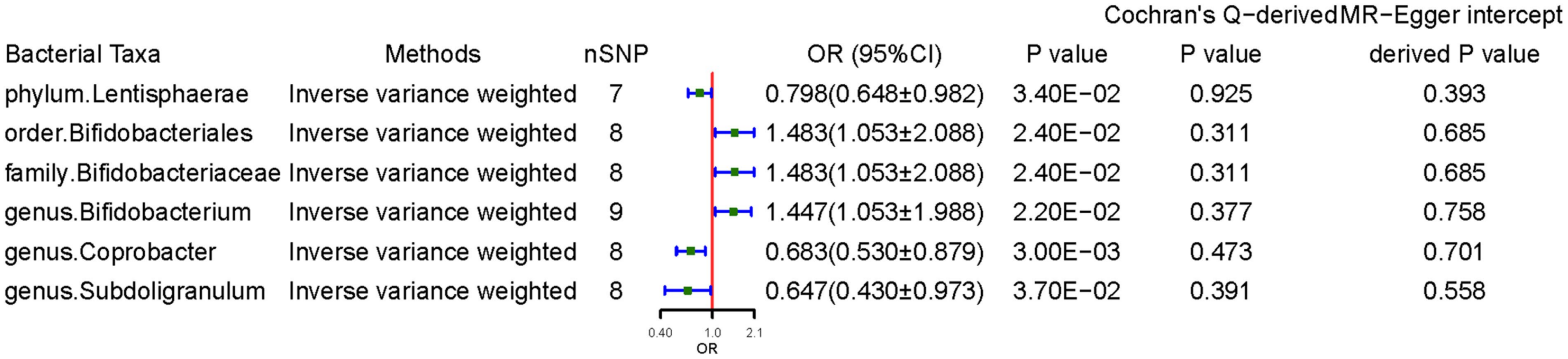
Causal effects of gut microbiota on celiac disease. OR, odds ratio; CI, confidence interval. Significant *p*-values after multiple-testing correction [phylum *p* = 5.56*10^−3^ (0.05/9), class *p* = 3.13*10^−3^ (0.05/16), order *p* = 2.50*10^−3^ (0.05/20), family *p* = 1.56*10^−3^ (0.05/32), and genus *p* = 4.20*10^−4^ (0.05/119)].

Figure 2 presents the significant IVW results (*p* < 0.05). At the phylum level, a higher genetically predicted *Lentisphaerae* level (odds ratio [OR] = 0.798, 95% confidence interval [CI]: 0.648–0.983, *p* = 0.034) was associated with lower CD risk. At the order level, *Bifidobacteriales* (OR = 1.483, 95% CI: 1.053–2.088, *p* = 0.024) was associated with higher CD risk. At the family level, *Bifidobacteriaceae* (OR = 1.483, 95% CI: 1.053–2.088, *p* = 0.024) was associated with higher CD risk. At the genus level, *Coprobacter* (OR = 0.683, 95% CI: 0.531–0.880, *p* = 0.003) and *Subdoligranulum* (OR = 0.647, 95% CI: 0.430–0.973, *p* = 0.037) was negatively associated with CD, suggesting a protective effect of these two bacteria. In contrast, genus *Bifidobacterium* (OR = 1.447, 95% CI: 1.054–1.988, *p* = 0.022) was associated with higher CD risk.

The weighted median method supported some association results. Notably, the SNPs in some bacterial taxa might have overlapped due to *Bifidobacterium* being a child taxon of *Bifidobacteriaceae*. The MR-Egger regression intercept revealed no evidence of horizontal pleiotropy (all intercepts, *p* > 0.05). The MR-Egger analysis yielded similar findings, which suggested no directional horizontal pleiotropy. Cochran’s Q test revealed no evidence of heterogeneity. However, MR power calculation demonstrated a significant (*p* < 0.05) causal effect with strong power (92%) of *Coprobacter* on CD.

### 3.3. Causal effects of metabolites on celiac disease

**Figure 3:**
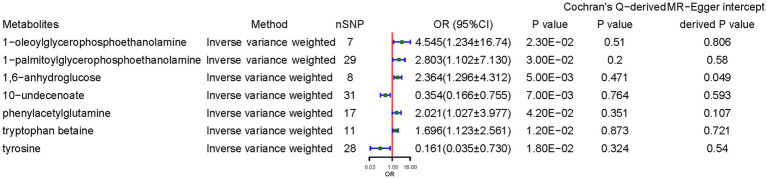
Causal effects of metabolites on celiac disease. OR, odds ratio; CI, confidence interval. Significant estimate is defined as IVW-derived *p* < 1.03*10^−4^ (0.05/486).

Genetically predicted higher concentrations of 1-oleoylglycerophosphoethanolamine (OR = 4.545, 95% CI: 1.234–16.741, *p* = 0.023), 1-palmitoylglycerophosphoethanolamine (OR = 2.803, 95% CI: 1.102–7.130, *p* = 0.030), 1,6-anhydroglucose (OR = 2.364, 95% CI: 1.296–4.312, *p* = 0.005), PAGln (OR = 2.021, 95% CI: 1.027–3.977, *p* = 0.042), and tryptophan betaine (OR = 1.697, 95% CI: 1.123–2.561, *p* = 0.012) were associated with higher CD risk. 10-Undecenoate (OR = 0.354, 95% CI: 0.166–0.755, *p* = 0.007) and tyrosine (OR = 0.161, 95% CI: 0.035–0.730, *p* = 0.018) were protective factors against CD. Sensitivity analyses deemed the results reliable without pleiotropy. MR power calculation demonstrated a significant causal effect (*p* < 0.05) with strong power (>80%) of 1-oleoylglycerophosphoethanolamine, 1-palmitoylglycerophosphoethanolamine, 1,6-anhydroglucose, 10-undecenoate, PAGln, and tryptophan betaine on CD (Figure 3).

### 3.4. Causal effects of celiac disease on gut microbiota

**Figure 4:**
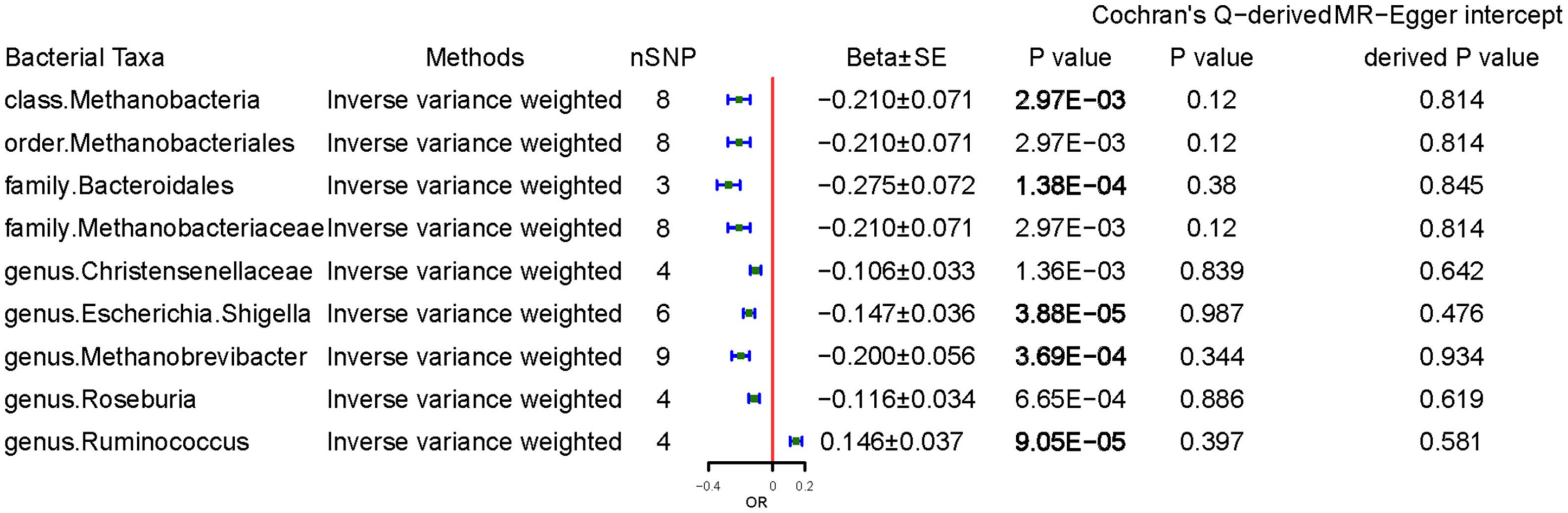
Causal effects of celiac disease on gut microbiota. Beta, The effect size of the exposure on gut microbiota. SE, standard errors. Significant *p*-values were marked in bold after multiple-testing correction [phylum *p* = 5.56*10^−3^ (0.05/9), class *p* = 3.13*10^−3^ (0.05/16), order *p* = 2.50*10^−3^ (0.05/20), family *p* = 1.56*10^−3^ (0.05/32), and genus *p* = 4.20*10^−4^ (0.05/119)].

We examined the causal effects of CD on gut microbiota. Sixty-four SNPs were associated with gut bacteria traits with an F-statistic threshold of >10. CD was causally and significantly associated with lower class *Methanobacteria* (per 1-unit odds ratio: Beta ± SE, −0.210 ± 0.071, *p* = 2.97 × 10^−3^), family *Bacteroidales* (−0.275 ± 0.072, *p* = 1.38 × 10^−4^), genus *Escherichia Shigella* (−0.147 ± 0.036, *p* = 3.88 × 10^−5^), genus *Methanobrevibacter* (−0.200 ± 0.056, *p* = 3.69 × 10^−4^), and higher *Ruminococcus* (0.146 ± 0.037, *p* = 9.05 × 10^−5^) levels per 1-unit higher log odds (Figure 4). The results from the other sensitivity methods are listed in Supplementary Table S5.

### 3.5. Causal effects of celiac disease on metabolites

**Figure 5:**
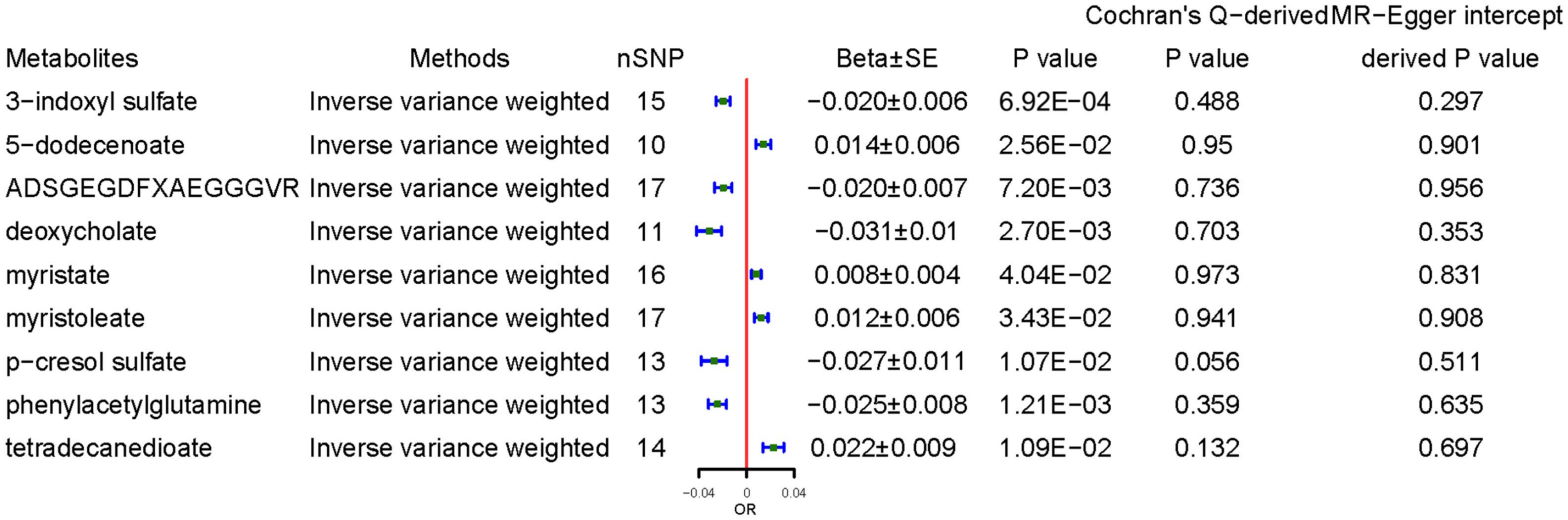
Causal effects of celiac disease on metabolites. Beta, The effect size of the exposure on gut microbiota. SE, standard errors. Significant estimate is defined as IVW-derived *p* < 1.03*10^−4^ (0.05/486).

CD demonstrated a positive causal association with 5-dodecenoate (0.014 ± 0.006, *p* = 0.025), myristate (0.008 ± 0.004, *p* = 4.04 × 10^−2^), and myristoleate (0.012 ± 0.006, *p* = 3.43 × 10^−2^), and tetradecanedioate (0.022 ± 0.009, *p* = 1.09 × 10^−2^). By contrast, CD demonstrated a negative causal association with concentrations of 3-indoxyl sulfate (3-IS) (−0.020 ± 0.006, *p* = 6.92 × 10^−4^), ADSGEGDFXAEGGGVR (−0.020 ± 0.007, *p* = 7.20 × 10^−3^), deoxycholate (−0.031 ± 0.010, *p* = 2.70 × 10^−3^), *p*-cresol sulfate (−0.027 ± 0.011, *p* = 1.07 × 10^−2^), and PAGln (−0.025 ± 0.008, *p* = 1.09 × 10^−2^) (Figure 5). The results from the other sensitivity methods are listed in Supplementary Table S7.

## 4. Discussion

The intestinal flora is considered a potential CD risk modulatory factor by interacting with metabolites and/or the host immune system (Valitutti et al., 2019). Microbiome and metabolomics studies over the past decades have greatly enhanced our understanding of the CD pathogenic mechanism (Caminero and Verdu, 2019; Leonard et al., 2020). Most documentation on the roles of the microbiome and metabolome in disease was from case–control studies aimed at determining the alterations that can be associated with specific diseases. Such studies can indicate associations but not causal relationships. Researchers are increasingly using MR to infer credible causal relationships between risk factors and disease outcomes. Here, we revealed suggestive associations between *Lentisphaerae*, *Bifidobacterium*, *Coprobacter*, and *Subdoligranulum* with CD. The metabolites 1-oleoylglycerophosphoethanolamine, 1-palmitoylglycerophosphoethanolamine, 1,6-anhydroglucose, PAGln, tryptophan betaine, 10-undecenoate, and tyrosine were also related to CD risk.

*Bifidobacterium* is the major microbe that primarily colonizes the human gut and is believed to influence the development of multiple autoimmune diseases. Olivares et al. reported that breastfed infants at genetic risk of CD had a decreased abundance of *Bifidobacterium* species in their feces (Olivares et al., 2015), suggesting its protective effect, while opposite results were reported for adult patients (Nistal et al., 2012). Recent studies demonstrated that *Bifidobacterium*-based probiotic interventions effectively delayed CD progression by reducing tumor necrosis factor-α production (Klemenak et al., 2015). Contrastingly, Smecuol et al. (2013) reported opposite observations. In our study, an increased relative abundance of *Bifidobacterium* was causally linked to the risk of CD, indicating its detrimental effect on CD. Several factors may have contributed to the inconsistent results as described above, on the one hand, considering that different bifidobacterium strains have different effects on the disease, for example, a particular bacterial group CMS-H004 may aggravate intestinal damage, while *B. breve* BR03 and B632 may alleviate intestinal damage. We hypothesized that bifidobacterium abundance would affect outcomes. Perhaps low abundance of probiotics can improve disease outcomes, but when abundance is high, it can aggravate disease damage. On the other hand, an increase in the relative abundance of Bifidobacteria will necessarily decrease the relative abundance of other probiotics, thereby weakening the beneficial effects of other probiotics. A more specific level, such as species, is crucial to provide more precise clinical guidance and direct mechanism research.

The phylum *Lentisphaerae* was proposed in 2004 after the isolation of two marine strains (Cho et al., 2004) and is a member of the Planctomycetes–Verrucomicrobia–Chlamydiae superphylum. Decreased abundance of *Lentisphaerae* was observed in patients with post-traumatic stress disorder (Hemmings et al., 2017), multiple sclerosis (Castillo-Álvarez et al., 2021), and autoimmune hepatitis (Kodio et al., 2019; Lou et al., 2020), while an increased abundance of this strain was observed in *Blastocystis*-colonized children (Kodio et al., 2019) and rosacea patients (Chen et al., 2021). In this study, a greater abundance of *Lentisphaerae* (OR = 0.798, 95% CI: 0.648–0.982, *p* = 0.034) was significantly related to lower CD risk. However, similar results have not been reported between the abundance of *Lentisphaerae* and risk of CD. Therefore, such results should be treated with caution. *Subdoligranulum* is a butyrate producer that is essential for gut health (Chassard et al., 2014), where it affects patients with necrotizing enterocolitis by influencing butyrate production (Lin et al., 2021). *Subdoligranulum* was significantly decreased in patients with chronic spontaneous urticaria and had potential diagnostic value for such patients (Liu et al., 2021). *Subdoligranulum* was also decreased in participants with food allergy (Abdel-Gadir et al., 2019). Abundances of *Subdoligranulum* species were markedly decreased in patients with inflammatory bowel disease (IBD) and extensively associated with concentrations of IBD-linked metabolites, which may lead to new possibilities for intervention (Sokol et al., 2008). In the present study, *Subdoligranulum* had a protective effect against CD. Nevertheless, the detailed mechanism remains to be illustrated in further studies. The reverse MR analysis revealed that CD was causally associated with *Methanobacteria*. The Methanogen archaea include *Methanosphaera stadtmanae*, *Methanobrevibacter smithii*, and *M. oralis* microbiota, which form a syntrophic relationship with other microbiota (Dridi et al., 2009). *M. smithii* prevalence in the intestine and gastrointestinal disorders are apparently linked (Aminov, 2013; Ghavami et al., 2018). However, no study stating its role in celiac disease has yet been reported.

Microbe-derived metabolites are crucial in host–microbe interactions. In the present study, 10-undecenoate and tyrosine were protective factors against CD. Previous MR analysis determined that 10-undecenoate shared an important positive causal relationship with rheumatoid arthritis (Yuan et al., 2022). However, no clinical or experimental studies supported the claim that CD affects blood 10-undecenoate levels. In a sense, this research provides a theoretical basis for follow-up research. Tyrosine is an essential amino acid that participates in host–microbiota crosstalk. Evidence from the other studies identified tyrosine levels were decreased after weight loss, indicating that it is a body mass index-associated amino acid (Geidenstam et al., 2017). Furthermore, tyrosine was associated with insulin resistance (Hellmuth et al., 2016) and the level of tyrosine was identified as a strong predictor of type 2 diabetes mellitus incidence (Tillin et al., 2015). It has been showed previously that gut bacteria metabolize tyrosine to *p*-cresol phenol sulfate and tyramine, which decreases intestinal epithelial cell viability and intestinal integrity (McCall et al., 2009). Interestingly, however, significantly lower tyrosine levels was observed in potential CD patients compared with CD patients (Upadhyay et al., 2022). Hence, Future validation is needed in cohort with more samples. PAGln was described as a gut microbiota metabolite fermented from dietary phenylalanine (Poesen et al., 2016). The microbial *porA* gene converts dietary phenylalanine into phenylacetic acid, with subsequent host generation of PAGln (Nemet et al., 2020). An elevated PAGln level may put a person at greater risk for acute myocardial infarction and type 2 diabetes (Poesen et al., 2016). Our results provided clinical evidence that the increased blood PAGln levels were also causally associated with CD occurrence.

3-IS is an important intercellular signal in microbial communities that is an indole metabolism byproduct produced by tryptophanase-expressing bacteria (Lee and Lee, 2010). Low urinary 3-IS levels were associated with higher transplant-related mortality and a worse overall survival rate in bone marrow-transplanted patients (Weber et al., 2015). Another study reported that during renal failure, high 3-IS levels were detected in patients with chronic kidney disease and cardiovascular disease (Vanholder et al., 2014). Moreover, it was previously found that 3-IS promoted the differentiation of anti-inflammatory and immunosuppressive dendritic cells (Ghimire et al., 2018). We assumed that the increased 3-IS levels might aggravate the inflammatory responses in patients with CD. Therefore, more in-depth investigations are necessary to determine the mechanisms by which metabolites promote CD occurrence.

García-Santisteban et al. (2020) and Xu et al. (2021) performed MR analyses to explore the causal relationships between gut microbiota and CD. Our study differed from theirs in two aspects. First, the identification of a causal relationship between metabolism and CD in the present study enabled a more comprehensive and integrated analysis of CD etiology. Second, compared to the study by García-Santisteban et al. (2020) which used gut microbiota data with a relatively small sample size (*N* = 1,126 twin pairs), we used microbiota data with a much larger sample size (*N* = 18,340) and more stringent IV screening criteria. Nevertheless, our study has several limitations. First, we failed to identify overlapping participants in the exposure and outcome of the GWAS summary data. Second, the bacterial taxa were not at a more specialized level, such as species or strain level. Third, the majority of participants in the GWAS summary data used in this study had European ancestry, but a small percentage of gut microbiota data was from other ethnicities, which might have resulted in the deviation of statistical results. Finally, owing to the limited variance explained by the SNPs or the limited sample size of the GWAS results, some of our MR analyses did not have sufficient power to detect small effects. Therefore, the results of the present study provide suggestive evidence of association. Given the limitations of the GWAS data, prospective studies are necessary to explore the underlying mechanisms in order to develop effective and feasible treatment strategies.

## 5. Conclusion

In conclusion, our findings supported the hypothesis that alterations in the abundance of gut microbiota and concentrations of metabolites are causally linked to the risk of CD. Differences in diet, habits, and lifestyle may contribute to individual susceptibility. Moreover, we identified the specific bacterial taxa and metabolites engaged in CD development. Nevertheless, the mechanisms behind these findings warrant further investigation.

## Data availability statement

The original contributions presented in the study are included in the article/Supplementary material, further inquiries can be directed to the corresponding author.

## Author contributions

TL designed the research and drafted the manuscript. TL, YF, CW, and TS collected and analyzed the data. AA and MZ performed the literature search. FG supervised the study. All authors were involved in writing the manuscript and contributed to the article and approved the submitted version.

## Funding

This study was supported by the Natural Science Foundation of Xinjiang Uygur Autonomous Region (Nos. 2021D01C200 and 20200103).

## Conflict of interest

The authors declare that the research was conducted in the absence of any commercial or financial relationships that could be construed as a potential conflict of interest.

## Publisher’s note

All claims expressed in this article are solely those of the authors and do not necessarily represent those of their affiliated organizations, or those of the publisher, the editors and the reviewers. Any product that may be evaluated in this article, or claim that may be made by its manufacturer, is not guaranteed or endorsed by the publisher.

## Footnotes

^1^ http://cnsgenomics.com/shiny/mRnd/
